# How countries cope with competing demands and expectations: perspectives of different stakeholders on priority setting and resource allocation for health in the era of HIV and AIDS

**DOI:** 10.1186/1471-2458-12-1071

**Published:** 2012-12-11

**Authors:** Françoise Jenniskens, Georges Tiendrebeogo, Anne Coolen, Lucie Blok, Seni Kouanda, Fuseini Sataru, Andriamampianina Ralisimalala, Victor Mwapasa, Mbela Kiyombo, David Plummer

**Affiliations:** 1 Royal Tropical Institute, Amsterdam, The Netherlands; 2 Biomedical and Public health Department, Institute of Research in Health Science (IRSS), Ouagadougou, Burkina Faso; 3 Centre for Health and Social Services, Accra, Ghana; 4 Department of Community Health, National Institute of Public and Community Health (INSPC), Antananarivo, Madagascar; 5 College of Medicine, University of Malawi, Blantyre, Malawi; 6 Ecole de Santé Publique, Kinshasa, Democratic Republic of Congo; 7 James Cook University & Queensland Health, Townsville, Australia

**Keywords:** HIV and AIDS, Health systems strengthening, Priority setting, Needs and priorities, Sector-wide approaches, Sub-Saharan Africa

## Abstract

**Background:**

Health systems have experienced unprecedented stress in recent years, and as yet no consensus has emerged as to how to deal with the multiple burden of disease in the context of HIV and AIDS and other competing health priorities. Priority setting is essential, yet this is a complex, multifaceted process. Drawing on a study conducted in five African countries, this paper explores different stakeholders′ perceptions of health priorities, how priorities are defined in practice, the process of resource allocation for HIV and Health and how different stakeholders perceive this.

**Methods:**

A sub-analysis was conducted of selected data from a wider qualitative study that explored the interactions between health systems and HIV and AIDS responses in five sub-Saharan countries (Burkina Faso, the Democratic Republic of Congo, Ghana, Madagascar and Malawi). Key background documents were analysed and semi-structured interviews (n = 258) and focus group discussions (n = 45) were held with representatives of communities, health personnel, decision makers, civil society representatives and development partners at both national and district level.

**Results:**

Health priorities were expressed either in terms of specific health problems and diseases or gaps in service delivery requiring a strengthening of the overall health system. In all five countries study respondents (with the exception of community members in Ghana) identified malaria and HIV as the two top health priorities. Community representatives were more likely to report concerns about accessibility of services and quality of care. National level respondents often referred to wider systemic challenges in relation to achieving the Millennium Development Goals (MDGs). Indeed, actual priority setting was heavily influenced by international agendas (e.g. MDGs) and by the ways in which development partners were supporting national strategic planning processes. At the same time, multi-stakeholder processes were increasingly used to identify priorities and inform sector-wide planning, whereby health service statistics were used to rank the burden of disease. However, many respondents remarked that health system challenges are not captured by such statistics.

In all countries funding for health was reported to fall short of requirements and a need for further priority setting to match actual resource availability was identified. Pooled health sector funds have been established to some extent, but development partners′ lack of flexibility in the allocation of funds according to country-generated priorities was identified as a major constraint.

**Conclusions:**

Although we found consensus on health priorities across all levels in the study countries, current funding falls short of addressing these identified areas. The nature of external funding, as well as programme-specific investment, was found to distort priority setting. There are signs that existing interventions have had limited effects beyond meeting the needs of disease-specific programmes. A need for more comprehensive health system strengthening (HSS) was identified, which requires a strong vision as to what the term means, coupled with a clear strategy and commitment from national and international decision makers in order to achieve stated goals. Prospective studies and action research, accompanied by pilot programmes, are recommended as deliberate strategies for HSS.

## Background

Over time, a rich rhetoric has emerged in relation to priority setting in the health sector [1-6]. Some analysts advocate that priorities should be based on epidemiological data. For example, despite its limitations, the calculation of the Disability Adjusted Life Years (DALY)^a^ by the World Health Organization (WHO) was an attempt to rationalise priority setting and provide comparable data on international disease burdens [7-9]. Others argue that community consultation and other bottom-up approaches for priority setting are essential, while some individuals criticise the current system in which, they argue, agendas are predominantly donor driven.

Since its emergence, the HIV and AIDS epidemic has disproportionally affected African countries which were already facing a multitude of problems including weak governance, widespread poverty, conflict, and natural disasters. In 2001 the United Nations declared HIV and AIDS to be an international crisis, requiring an extraordinary response [10]. In the same year the Commission on Macroeconomics and Health and the Commission on HIV and AIDS and Governance concluded that the lack of political will to sufficiently increase spending on health at sub-national, national and international levels was perhaps the most critical barrier to improved health in low-income countries, exposing the need to both remove financial constraints and ensure strategic investment aimed at increasing health sector capacity [11].

Over the past decade, global resources for HIV increased dramatically through public funding such as the Global Fund to Fight AIDS, Tuberculosis and Malaria (GFATM), the United States President's Emergency Plan For AIDS Relief (PEPFAR), bilateral and multilateral aid and private sources, such as Global Health Initiatives and private benefactors [12]. Between 1998 and 2007, funding for HIV prevention, treatment and support increased from 5.5% to 47.2% of all donor health investment. Over the same period, while total aid for health tripled, funding for health systems strengthening (HSS) fell from 62.3% to 23.9% of total funding, resulting in the stagnation of HSS support [13]. Overall, the HIV epidemic prompted an extraordinary response in terms of funding, speed and scale, and is often portrayed as overfunded and reinforcing vertical, disease-specific approaches [14-18].

Drawing on the United Nations General Assembly Special Session on HIV (UNGASS) and the National AIDS Spending Assessment (NASA) reports, a comparison between the per capita expenditure on HIV and AIDS and health in the five study countries shows that Malawi has the highest ratio of HIV against health spending (0.14:1); Burkina Faso, Madagascar, the Democratic Republic of Congo (DRC) and Ghana were much lower with a ratio in the range of 0.02-0.08: 1 (see Table 1).

**Table 1:** Comparison of per capita expenditure on HIV and AIDS and health in the five study countries

**Ratio HIV/Health**	**2006**	**2008**	**2009**
Burkina Faso	0.04	0.05	0.06
DRC	0.04	0.08	NA
Ghana	0.02	0.02	NA
Madagascar	0.03	0.01	NA
Malawi	NA	0.14	NA

Data sources: [19-27].

By the end of 2009, 5.25 million people worldwide were receiving antiretroviral treatment (ARV), the majority of whom (3.9 million) were living in sub-Saharan Africa. Moreover, a reduction of about 25% was observed in the incidence of new HIV infections across 22 sub-Saharan countries [28]. Yet although impressive achievements have been made in the HIV and AIDS sector, there is a consensus that health systems in African countries can barely cope with increasing demands and that the MDGs are likely to remain elusive unless system-wide barriers are addressed [29,30].

Despite global consensus that there is a health system crisis, views on how to deal with multiple burdens of disease in resource-poor settings vary [29-34]. While priority setting is essential, this is a complex and multifaceted process. The continued struggle for scarce resources within the health sector has led to debates among international experts concerning the exceptionality of AIDS and how national health policy makers may have different perceptions of priority needs [35]. The discussion has further evolved around balancing the needs of an effective HIV response with investment in other disease-specific programmes and the ways in which funding strategies could contribute to wider HSS efforts [34,36-39]. Little is known about how either health planners or communities perceive health needs and priorities, and how disease and overall programming priorities are set in reality; the actual processes leading to resource allocation are not well documented.

In the light of the situation at the time of the study, this paper explores the following questions: 1) how do different stakeholders understand health priorities? 2) how are health priorities set in practice? 3) how are decisions made about resource allocation? 4) how do different stakeholders perceive resource allocation to HIV and AIDS as compared to other health priorities?

This inquiry forms part of a larger qualitative multi-country case study conducted in 2010 in five sub-Saharan countries, which aimed to explore synergies between HIV programmes and HSS efforts [14-18,40].

## Methods

The study used qualitative methodologies to elicit the perceptions of different stakeholders. Countries were sampled purposively to include a diversity of settings and contexts. Selection criteria included countries with low, medium, and higher levels of HIV; different geographical regions of sub-Saharan Africa (west, central and southern); variation in country contexts (post-conflict versus more politically stable; different experiences with sector-wide approaches, differences in domestic versus international funding for HIV and health); and the interest of national authorities in participating in the study. The countries selected were Burkina Faso (medium level HIV; francophone West Africa; some experience with sector-wide programming; high donor dependency; relatively stable), Ghana (medium level HIV; anglophone West Africa; strong experience with sector-wide programming; low level of donor dependency; relatively stable), Madagascar (very low HIV prevalence; francophone southern Africa; little external investment; sector-wide approach in its infancy; post-conflict setting), the DRC (medium level HIV prevalence; francophone central Africa; severely under-resourced HIV and health sector; no sector-wide approach; conflict situation; high donor dependency) and Malawi (high HIV prevalence; anglophone southern Africa; strong experience with sector-wide approaches; relatively stable; high donor dependency).

Ethical approval for this study was obtained from the Royal Tropical Institute (KIT) research ethics committee as well as from ethical review committees in the respective countries.^b^ All respondents were asked to sign a consent form.

In each country two districts were purposively selected based on discussions with national advisory groups, which included representatives of the Ministry of Health, the National AIDS Council, civil society groups and national research institutes. The aim was to include two districts that would provide contrasting examples of the interaction between HIV and AIDS programming and the wider health system. In each district a minimum of six health facilities and three communities (one in a remote rural area with little access to a health facility, one in a rural setting but with easy access to services and one in the district capital) were included. Most interview respondents were recruited on the basis of their specific position and knowledge about the issues under study and the response rate was high. Community leaders and civil society groups identified participants for community-based discussions.

The studies were carried out by teams of experienced researchers from established research institutes in each country, supported by a team of international researchers. Data collection methods included desk reviews, interviews and focus group discussions (FGDs). Semi-structured interviews were conducted with key informants (government, development partners and civil society representatives) at national, district, health facility and community level. Respondents were first asked to identify and rank their perceived health priorities. The interviews then explored priority setting processes, national health planning processes, resource allocation, donor coordination, and harmonisation and alignment between government and development partners for aid effectiveness.

Up to four FGDs were held in each selected community with women, men and youth, and community volunteers or community health committee representatives. The groups were also asked to identify and rank their perceived health priorities and needs and the discussions focused on these.

Most interviews and FGDs were audio recorded and translated from local languages (such as Mooré, Lingala, Chichewa, Malagasy) into French or English, where required. All interviews from Malawi, Ghana and Burkina Faso were transcribed, coded and analysed. In Madagascar not all transcripts were translated. Most data were coded and analysed using ATLAS.ti software (version 6.2) for qualitative data analysis. In total, 543 interviews and 45 FGDs were conducted in the five countries. Out of these, 258 interviews and 45 FGDs were transcribed and coded until key themes and variables did not elicit new information. Some interviews were not properly audio recorded and some FGDs were not translated so these data were not used. Field notes taken by researchers during four interviews from the DRC (that were not recorded due to technical problems with the recorders) were also used as data. See Table 2 for a summary of the total number of interviews and FGDs that were coded and analysed by country (the unrecorded data from DRC which were used are presented in brackets).

**Table 2:** Number of interviews and FGDs by country and level coded for analysis

**Country**	**Burkina Faso**	**DRC**	**Ghana**	**Malawi**	**Madagascar**	**All countries**
Interviews Community Leaders and Members	7		24	6	5	42
Health Workers	21	11 (+3)	10	13	8	63
District Level Governmental and NGOs	10	6	12	16	7	51
National Government Level	14	8 (+1)	10	22	5	59
Development Partners	7	2	4	9	6	28
National level NGOs	2	2	2	8	1	15
FGD Community Leaders and Members	18	2	13	7	5	45
Total	79	31 (+4)	75	81	37	303(+4)

Data analysis was carried out in two stages. In-country analysis and validation workshops were conducted with the research teams that conducted the study, together with the international researchers. Through a preliminary analysis using key word searches on the transcripts and interview notes, key emerging themes were identified. This was followed by full coding of the transcripts and notes and further analysis of individual country data, using ATLAS-ti software. The principal investigators from each country then participated in a cross-country analysis workshop held in Amsterdam with the international researchers.

### Limitations of the study

Because only two districts in each country were studied, the findings are not necessarily representative of the situation in other districts and cannot be extrapolated. Instead the study provides insight into the diversity of the perceptions and opinions of key stakeholders within and across the study countries.

Translation from national languages into French and English is likely to have resulted in a loss of some meaning and detail. Further, there were variations in the way questions on priority setting were formulated by different field researchers, which may have influenced responses.

## Results

### What is the perception of health priorities amongst different stakeholders?

Study participants were first asked to list and then rank health priorities based on their relative importance. Two patterns of response were identified: 1) priorities articulated as significant health problems and diseases, and 2) priorities framed as gaps in health services that required strengthening of the overall health system.

#### Priorities stated in terms of diseases and specific health needs

Most respondents across the five countries ranked either malaria or HIV as priority number one or two.

There are various diseases … we can say malaria is the first one, also there are some diseases which come because of HIV and AIDS, therefore there are diseases like diarrhoea, also TB [tuberculosis] and malnutrition, another one is pneumonia.

Community member FGD participant, Malawi

According to those who ranked HIV and AIDS as the first priority, this was mostly due to the severity of the disease and the difficulties in treating it. Malaria was considered more dangerous in terms of mortality and spontaneous abortions, as explained below.

Malaria is the first health problem in our community, both adults and children suffer from it and mortality due to malaria is very high. Then, comes AIDS, which creates anxiety in our community, and lastly minor ailments such as cough and stomach pains.

Community leader, key informant interview, Burkina Faso

It [AIDS] is a bad disease, if you catch it, it's hard to heal. In the case of malaria one can be treated right away and totally recover, but AIDS is difficult to treat, that's why I ranked [it] first.

Community member FGD participant, Burkina Faso

The level of priority assigned to HIV and AIDS differed amongst the five countries, and did not always relate to national HIV prevalence levels. In both Malawi (which has a high HIV prevalence), and Burkina Faso and DRC (countries with much lower HIV prevalence), most respondents mentioned HIV and AIDS as one of the two main health priorities. The reasons provided for prioritising HIV and AIDS were concerns about the seriousness of the disease and the broader negative social and economic effects.

We are more concerned with its consequences for our children. HIV kills young people in their prime of life and leaves communities with orphans.

Community member FGD participant, Burkina Faso

Even in Madagascar, the country with the lowest HIV prevalence amongst the five study countries, the majority of respondents also mentioned HIV and AIDS as a priority. Given the seriousness of the disease and its long-term impacts, a number of respondents at all levels felt that it was important to focus on HIV prevention despite the current low prevalence:

AIDS? Ha! It is a priority. People hear about it all the time. In fact, AIDS information campaigns have become routine during community meetings …. Yes, it’s a priority for the population. Awareness-raising campaigns explain that AIDS impact is limited in Madagascar and the disease is not visible in our midst.

Community leader, key informant interview, Madagascar

AIDS has straight away been included because it was initially feared it would be devastating. Fortunately, it has been contained and has now stabilised. And the most surprising is that so far we do not understand why AIDS has not increased that much.

National level stakeholder, Madagascar

In contrast, in Ghana the majority of the community and district level respondents -including health workers - did not see HIV and AIDS as a priority. In both districts included in the study (one with high and one with low HIV prevalence), people stated that HIV and AIDS was not a problem and did not deserve the level of attention it currently received. Respondents instead emphasised the need for greater investment in health services, clean water, and education.

I think the government should stop the AIDS programmes and use the money for more important things [such as] pipe-borne water, hospitals, buying exercise books and providing free education.

Community member FGD participant, Ghana

While such sentiments were particularly common amongst study participants at community level, most health planners and development partners at district and at national level in Ghana listed HIV and AIDS as a top priority.

Other priorities mentioned by most respondents in each country were diarrhoeal disease, acute respiratory infection (ARI), tuberculosis (TB), maternal health, childhood diseases and malnutrition. In addition, community members, health workers and district level respondents mentioned skin diseases and schistosomiasis.

The source of our drinking water is not good, some people contract schistosomiasis, that is blood in urine. Malaria is also common in this community. We also have other diseases like bilharzia, gonorrhoea and hypertension. I must also mention that the sanitary conditions in this community are very poor. There are no public toilets and people defecate in the river, which is the source of our drinking water. This has led to the outbreak of some of the illnesses that I have mentioned.

Community member, key informant interview, Ghana

Furthermore, it was noteworthy that in Ghana (and to some extent Madagascar, Malawi and Burkina Faso) respondents at community and district level sometimes mentioned chronic and lifestyle diseases such as hypertension, stroke and diabetes as important priorities. National level respondents and development partners less often mentioned these.

#### Priorities stated as health system challenges

Community members in rural areas in Ghana and Burkina Faso expressed serious concerns regarding health system-related constraints. For example, they mentioned problems in accessing health services (due to distance), non-availability of qualified staff, the unresponsiveness of available staff, and inadequate drug supplies and equipment. These concerns were often contextualised in relation to problems in access to other basic services (schools, electricity, clean water, etc.).

The distance from this place to the clinic is too long so when one is sick it takes a long time to get to the clinic for treatment and so people die on the way.

Community member FGD participant, Ghana

In this community we need a school very badly. Also we don’t have electricity, a clinic, and a good source of drinking water. For the health conditions, malaria and fever are very common especially with the children. HIV is not present in this community.

Community leader, key informant interview, Ghana

Across all five countries, respondents at district and national level (both government respondents and development partners) identified limited infrastructure, lack of (qualified) personnel, poor material and financial resources, inadequate supplies and weak planning and management capacity as key barriers in achieving better health.

We do not have enough human, material or financial resources for the future; therefore we are reduced to just do with what we have. Our health care system is not yet capable to plan for the future, that is to say, to start planning from now.

Development partner, national level, Madagascar

I think from the perspective of the Ministry… HRH [Human Resource for Health] stands out as the main area of the health system that requires strengthening. Then from there we have the other processes and systems like procurement and financial management.

Government representative, national level, Malawi

The broader health systems challenges, which were identified by national level health planners and development partners, were mostly seen as obstacles to achieving the MDGs.

I think there is some underlying problem, which we don’t talk too much about and we should talk about it today. The WHO and lots of other development partners are pretty convinced now that for any country to achieve the MDGs or any specific programme to achieve the MDGs, the strengthening of the six major pillars of what constitute a health system must be carried out. In Ghana, we have some work to do.

Development partner, national level, Ghana

Many respondents articulated system challenges from both user and planning perspectives and felt that health system strengthening (HSS) was of key importance to address these challenges.

### How are health priorities set in practice?

#### Multi-stakeholder processes in planning

Decision makers confirmed that priority setting, policy and strategy development were important processes that require multi-stakeholder involvement. In all countries national level policy makers described a bi-directional dialogue about priority setting and policy making. The development of national health strategies and plans was described as typically beginning with discussions between community constituencies and district level authorities about priority needs. Consultations were often conducted as multi-stakeholder meetings, allowing different interest groups to contribute and express their views. These discussions then feed into a chain of communication running from community levels up to the national level.

In general, when we look at health planning, I believe it is being done in a much more participatory way. It's like we are trying to involve all stakeholders…I think it's much more participatory and more multi-sectoral. The NGOs, the ministries, the technical and financial partners have been integrated, altogether.

Development partner, national level, Madagascar

All the various actors that we have in the field…all these actors are represented at health council levels. Already at each health facility level planning is even participatory; there is community involvement.

Government respondent, national level, Burkina Faso

#### Priority setting using epidemiological data

Many respondents stated that health priorities are derived from health statistics and the health management information system data from district level. As these are synthesised from daily service statistics, common morbidities in the communities are captured.

Our prioritization in relation to health will be based on the data we get from the GHS [Ghana Health Services] and the health facilities.

Government respondent, district level, Ghana

As a result of this, essential health packages (EHP) are designed which address priority diseases.

And in this EHP, we have got a list of diseases that we need to take care of. …As a ministry, we come up with one plan of action, addressing our common priorities…using the data we get from HMIS and the disease control programmes. So that you know which ones are our main causes of morbidity and mortality. And then we put most of the resources in areas where there are bigger problems.

Government representative, national level, Malawi

While in several of the study countries HIV and AIDS do not figure in the top five disease prevalence statistics, it is prioritised because of other reasons. Firstly, national level government respondents and development partners across all countries consistently made reference to the MDGs and reported how these goals were reflected in priority setting processes.

I think the main guiding policy for government, in terms of priorities, is the MDGs. So the Ministry’s mandate and government policies have come from the MDGs. HIV and AIDS is one of the six. So those are like the priority, not just solely for the Ministry.

Government respondent, national level, Malawi

Secondly, HIV and AIDS modelling at country level informs the way in which the epidemic spreads and predicts the potential impact of the disease. In Burkina Faso for example, national level government health officials characterised HIV and AIDS completely differently from other health problems.

Yes, malaria as I said is the first major health problem according to the official data from the Statistical Index… and generally, when you read the statistics, HIV paradoxically does not appear among the 10 leading causes of consultation in healthcare facilities in our country. Nevertheless I think HIV is even more than a health problem; it’s the range of social and economic consequences that it poses that makes AIDS a problem…. HIV testing data is derived from sentinel surveillance sites or from specific testing centres and therefore is not captured in the service statistics.

Government respondent, national level, Burkina Faso

### How are decisions made about resource allocation?

#### Sector-wide approaches

In terms of the policy environment, many countries have health sector development plans and have adopted Sector-wide Approaches (SWAp) in an attempt to better harmonise and coordinate efforts and facilitate different development partners to contribute to more effective planning and programming. The nomenclature varies across different countries but the underlying motivations and principles are the same. The level of implementation of SWAps differs depending on the country. In Ghana, the SWAp for health commenced in 1996 and has guided developments in the health sector ever since [41]. In Malawi, the SWAp for HIV [42] preceded the introduction of the SWAp for Health by one year despite the fact that discussions concerning SWAp first commenced in the health sector in the late nineties [43]. In Madagascar, there is a SWAp for HIV but not yet a SWAp for health [44]. Burkina Faso has developed sector strategic frameworks for HIV and AIDS and National Plans for Health Sector Development since 2001 [15]. The DRC has a National Health Policy, which emphasises key health priorities, and a sector-wide plan for HIV and AIDS [45].

We have had various types of funding mechanism. We used to have a system whereby donors pool their funding together but this was not basket funding, then we had the general budget support, with all these you will still have the earmarked funds. Then we came to the basket funding and some donors did not participate, next was the sector-wide approach and the composite budgetary support.

Government respondent, national level, Ghana

While respondents were positive about the development of sector-wide plans as an important mechanism for priority setting and harmonising activities, there was also some criticism about the influence of development partners.

If you look at the HIV/AIDS policies, they are influenced by donors… even [when developing] the policy governing the SWAp, the donors took a substantial amount of time trying to work with the government and influence the way things should be.

Government respondent, national level, Malawi

I think that for us in the health sector, because of the SWAps, our relationship with donors changed some time ago… it put us in the driving seat. Then, it also made the donors important in steering, in the cockpit. So, what that means is that we are in charge of steering, but the donors pull a lot of strings from the back. Sometimes, they can even twist your arm.

Government respondent, national level, Ghana

Funding decisions are made within the framework of national health plans and aim to respond to defined health priorities. However, all countries face limitations in the availability of financial resources to fund the entire national health plan.

We allocate our resources also in line with where our priorities fall. … It [the need] is more than what we have, but we are trying to deal with it with the resources available to us.

Government respondent, national level, Ghana

Most national level respondents considered support for sector budgets as the ideal situation. It strengthens country ownership and provides a means for development partners to contribute and align their support towards a country’s own self-identified needs and priorities.

If you look at the programme of work, it has six areas, but these are the ones we must fund. These are the priorities for this year and the donors agree. Of course there are donors in the sector who still earmark their funds for certain activities but that is within the concept of SWAp, other than everyone just coming individually into the playground.

Government respondent, national level, Malawi

#### Donor influence over resource allocation: positive and negative aspects

Given that national health budgets are insufficient, all study countries rely on development partner contributions. In addition, it was reported that these partners have an influence on which priorities get funded, both in terms of where money is allocated and the conditions attached. Many national level officials reported that resource allocation, and therefore what gets implemented, is ultimately determined by the availability of external funds. This is even more so in a failing state like the DRC, but was also identified elsewhere.

… Specific programmes supported by the international partners, coupled by the lack of contribution of the Congolese government, do indeed take advantage and dictate [their law] as the saying goes ‘the hand that gives is the one that dictates’… Most institutions depend on and comply with their own funding partners’ requirements, even if these are not in line with the strategic priorities of the Congolese government.

NGO respondent, national level, DRC

We are in a country, I want to say poor, but we are clinging to the donors. That's the reality, HIV [related donors] would come and say, I want to fund HIV, and since HIV is also identified [as a need] at the population level, what do you want us to do? We'll take the money. Tuberculosis [related donors] would come and say I will do TB; reproductive health, and so on … that's the problem.

National health planner, Burkina Faso

If funding is made available, we will never say “no” to it, whether it covers a priority need or not.

Government representative, national level, Ghana

Development partner funds are often accompanied by strict ring-fencing measures ensuring that their support is solely used for pre-defined priority areas. Some stakeholders welcomed this approach.

With all these we still had the earmarked funding and ring-fencing. For instance we ring-fence for family planning commodities. With ring-fencing, various people pay and it is used for the purchase of specific commodities. The NGOs, civil society and others normally would not have benefitted from the SWAp or government funding system.

Health NGO network chair, national level, Ghana

Some stakeholders also highlighted the positive side of development partners influencing priority setting. As development partners bring with them global evidence on good practice and evidence informed approaches, they can be instrumental in highlighting important, overlooked issues such as sexuality, human rights and the situation of marginalised groups, that otherwise might not feature in health sector programming.

People may overreact, they may be shocked, and they may be in denial and all that. Fine! If you should go out there and say that we should repeal the criminalization of those practices [Sex Workers and Men Who Have Sex With Men] in the Criminal Code, I’m not sure we are ready to do that. There is the need to put more focus on some of the Most-At-Risk Population (MARP) in terms of programming… there has been an improvement in using the evidence that is generated in ensuring that we set the right priorities.

Development partner respondent, national level, Ghana

There are also other funding mechanisms that allow for more flexibility, for example one international NGO supports the health system in one district in Malawi. Respondents from this district indicated that they have used this situation to reallocate resources and maximize the benefits of external investment across the entire health programme.

We, at our local level… we only get international donor money, which is one big envelope, but which we can allocate according to our local priorities … which we do together with the DHO (District Health Officer).

NGO respondent, district level, Malawi

### How do different stakeholders perceive resource allocation to HIV and AIDS compared to other health priorities?

#### Perception that HIV and AIDS investment skews priorities

All the study countries receive funds for HIV and AIDS, both for multi-sectoral and health sector response strategies. Many respondents were of the opinion that there was an imbalance between the funding available for HIV and AIDS programmes compared with overall funding for health. Some expressed the view that the high proportion of funding for HIV and AIDS results in this dominating other health priorities.

The influence of the HIV response in the health sector is that HIV takes over other health priorities and there is a growing feeling that planning in this area could use some work.

Government respondent, provincial level, DRC

In general, I think the amount of money coming in for HIV/AIDS is disastrous, it’s too big so it is dwarfing all kinds of other things. In general there is too much money and it is too verticalised.

Development partner, national level, Ghana

#### Positive impacts of HIV and AIDS investment

Others, however, did not believe that the available resources for HIV and AIDS had a major influence on government health expenditure. Furthermore, several respondents believed that the health system as a whole has benefited from responses to the disease.

HIV/AIDS has also come to save our system from eminent collapse. If we look at government funding sources, if you take away what the Global Fund brings in the area of HIV/AIDS, TB and malaria, our system would have collapsed by now.

Government respondent, national level, Ghana

## Discussion

This paper aims to explore how various stakeholders understand health priorities, how priorities are established in practice, their relationship with resource allocation, and perceptions of the funding of HIV and AIDS in relation to other health priorities. The findings presented relate to the situation in 2010, when the study took place. Since that time new developments in national and global health priority setting can be seen to have affected the discourse concerning synergies between HSS and HIV and AIDS. However, the issues raised by the study findings concerning health priority setting and resource allocation processes remain relevant.

Overall, in all study countries priority diseases mentioned by respondents include malaria, HIV and AIDS, diarrhoea, respiratory tract infections and TB. HIV and malaria generally constituted the top two ranked health problems and were often prioritised by community members. This could be related not only to the overall burden of disease but also to the risk of dying, the availability of effective treatment and the psychosocial and broader societal impacts (especially in the case of HIV and AIDS). Indeed, HIV prevalence rates did not seem to be the only reason for prioritising HIV, as in low HIV prevalence countries such as Burkina Faso, with a HIV prevalence of 1.6% [19] and Madagascar, where HIV prevalence is 0.13% [20], community members also attached high priority to this disease. However in Ghana, where HIV prevalence stands at 1.9% [24], community members and health workers were of the opinion that the importance of HIV and AIDS was highly overrated and more systemic priorities such as safe water, electricity, schooling and roads were highlighted.

Apart from specific disease priorities, respondents at community level in all five countries included broader health system challenges in their priority setting. These included access to higher quality health services and to other basic services such as clean water, sanitation, and electricity. This shows that to these stakeholders it is not only diseases that are important, but also the nature of current health services as well as broader contextual and development issues which impact on health and the functioning of the health system.

At the national level, health priorities were also articulated in terms of disease burdens, but more emphatically in terms of health systems challenges. Priority diseases at national level did not differ much from those mentioned by communities with the exception of Ghana where national-level respondents attached much more importance to HIV and AIDS.

Policy makers and development partners at national level voiced particular concern about systemic weaknesses, unavailability of resources and challenges in management and governance. In addition, they highlighted that without addressing these broader health system challenges none of the MDGs would be met.

As reported earlier, the DALY were introduced to establish the relative disease burden at a country level and were expected to assist health planners in defining priority areas. However, none of the study respondents referred to the use of DALY for setting priorities, instead service level statistics were reportedly used for this purpose. Such health statistics do not capture health system related needs or important contextual and development factors (a limitation that would, indeed, also apply using the DALY calculations).

Our study reveals that in most countries national policy makers and planners involve different stakeholders in the development of sector-wide policies, strategic and annual plans. These processes involve consultations at district and local government level and include civil society actors and groups, so as to take local priorities and needs into account. However, both health planners and development partners in all countries appeared to be highly influenced by the international health and development agenda, particularly the MDGs. For example, the diseases targeted by both the Global Fund to Fight AIDS, TB and Malaria and the MDGs were mentioned as a guiding framework for priority setting and, consequently, resource allocation.

The role of development partners in priority setting processes was perceived as both negative and positive, from the pushing of international or other external agendas to the introduction of new issues that may otherwise have been overlooked, such as rights-based approaches and inclusive planning for marginalised groups. But with policy makers facing numerous constraints, in particular insufficient health budgets, an important, intertwined dimension of priority setting relates to the availability of funding and the specific interest/s of development partners; a problematic situation which is not exclusive to the health sector [46].

As the data presented above reveal, resource allocation is negotiated between national and international players. However, regardless of the priorities stipulated in national health development plans, the programmes that receive donor funding are invariably those which conform to international donor priorities. It could furthermore be argued that a disease becomes prioritised when it has a high international profile.

Detailed, disaggregated financial data in terms of funding for specific diseases attributed by development partner and/or national funds were not available in the countries studied. However, the earmarking of international resources is a political choice and there was a general recognition amongst respondents that ‘who pays the piper, calls the tune’, which on the one hand could be attributed to weakness of national stewardship and/or to the emphatic influence of development partners during national health strategic planning and resource allocation processes.

Although sector wide policies are meant to facilitate country ownership and the pooling of funds, it was reported that not all development partners are willing to operate according to this approach. Ghana, Malawi and Burkina Faso stood out as examples where at least part of the funds for health and HIV and AIDS were pooled. In the DRC, a post-conflict country with a very limited national budget for health and few external resources, that are not pooled, managers struggle to find sufficient resources for their own programmes.

Many national level respondents felt that the availability of disease-specific funding for HIV and AIDS and other priority programmes influenced and distorted their priority setting during planning. The earmarking of funds for HIV and AIDS programmes was often seen as a constraint in addressing other health priorities, particularly health systems challenges, even though in Malawi, for example, funds from the GFATM had been used to strengthen the health workforce [17]. A common perception among many respondents is that a “crowding out effect” of other health priorities by HIV and AIDS has occurred, although this is not confirmed if actual per capita health expenditure is compared to per capita HIV and AIDS expenditure. While Malawi shows the highest HIV/Health expenditure ratio, the highest levels of resentment were found in Burkina Faso, the DRC and Ghana, countries with the lowest HIV/health expenditure ratio.

In order to understand current resource allocation processes, there is a need to look deeper into the power over resources, which is held by different actors. Priority setting and particularly the process of resource allocation and the flow and management of money, reveal patterns of power relations. At the same time, if we specifically analyse the resources allocated to fight HIV and AIDS, there are other issues that need to be taken into consideration. HIV disproportionately affects marginalised populations and touches deeply on taboo issues, which leads to stigmatisation of people living with the disease. One reasoning behind the earmarking of funds could be that it is to prevent it being siphoned off to other, less taboo, issues and that it is controlled to make sure that human rights are not sacrificed to the politics of the day.

The call for increasing focus and associated funding levels for HSS, has led to a growing interest by major donors and international programmes (GFATM, PEPFAR, World Bank, Department for International Development (DFID, UK), Global Alliance for Vaccines and Immunisation (GAVI), etc.) in strengthening health systems alongside supporting established priority programmes [47]. For example, at its inception the GFATM focused on only three target diseases and shifted over time to include public and community health systems strengthening components, joining forces with GAVI and the World Bank in the Health Systems Funding Platform.

Even though there was recognition amongst study participants that a more explicit strategy for HSS was needed, national respondents felt that programmes are still directly linked to international agendas and/or disease-specific priorities, hence HSS is only supported where it strengthens specific programme outcomes rather than reflecting a broader vision on how a generally stronger system could meet defined priorities programmes in a more equitable and effective manner [10-13]. At the same time, respondents expressed a strong conviction that without a profound rethink of the health delivery system most major health programmes will not reach their objectives and none of the MDGs will be achieved.

## Conclusions

In the countries in which the study was implemented, most respondents viewed HSS as a necessary condition to meet the MDGs. Stakeholders’ and community members’ perceptions concerning the relative importance of specific diseases and health needs did not differ markedly. However, implementing the transformations needed to meet either disease specific or broader health systems needs is constrained by the limited margin of manoeuvre enjoyed by national policy and programme planners. Both global health perspectives and priorities and development partners’ political processes for funding distort autonomous programming for health at a national level and still exert a strong influence over priority setting and resource allocation in all five countries.

A need for more comprehensive health system strengthening (HSS) was identified, which requires a strong vision as to what the term means, coupled with a clear strategy and commitment from national and international decision makers in order to achieve stated goals for improvement. Prospective studies and action research, accompanied by pilot programmes, are recommended as deliberate strategies for HSS.

This study showed that many health planners see sector-wide funding as the preferred approach, enabling the design and implementation of efficient, country-owned health plans and programme work. This requires both pressure on development partners to release some of the strings attached to development aid, as well as increased transparency and strengthened national governance and accountability mechanisms at country level to safeguard (amongst other things) human rights and equity in national planning processes and resource allocation.

## Endnotes

^a^Disability Adjusted Life Years (DALY) were developed as a measurement unit for the burden of disease. The burden of disease, and an understanding of the risk factors that cause this, can be used by policy makers for health policy and planning in conjunction with cost-effectiveness studies in order to compare the intended impact of policies in relation to the implied cost associated with a reduction of the burden of disease.

^b^République Démocratique de Congo, Ecole de Sante Publique, Comité d’éthique. Numéro: ESP/CE/026/2010

Ghana Health Service Ethical Review Committee. Number: GHS-ERC:12/7/09

République Madagascar. Ministère de la Sante et du planning familial/Secrétariat General. Numéro: 413SANPF/SG

Ministère de la Sante, Burkina Faso. Comité d’éthique pour la recherché en santé. Numéro: 2010-0010/MS/MESSRS/CERS

Malawi. College of Medicine Research and Ethics Committee. Number: S11

Research Ethics Committee of the Royal Tropical Institute. Number: S11, 9th August 2009

## Competing interests

The authors declare that they have no competing interests.

## Authors’ contributions

FJ led the conceptualisation of the study and participated in the cross-country analysis of the multi-country study and provided technical assistance to the data collection in Malawi and Madagascar. She conceptualized and coordinated the development of the framework of analysis, conducted the overall literature review, analysed data from all five countries and drafted this article. GT contributed to the conceptualisation and cross-country analysis of the study, provided input to the framework for analysis, provided technical assistance to data collection in DRC and Burkina Faso, analysed data from all five countries and wrote up the results. AC provided input to the framework for analysis, provided technical assistance to the data collection in DRC and Madagascar, analysed data from all five countries and assisted in the writing process. LB participated in the conceptualisation and coordination of the study, led the overall cross-country analysis, provided technical guidance to data collection and analysis in Ghana, Malawi and Madagascar and provided input to the framework for analysis for and drafting of this article. VM led the data collection and analysis in Malawi and reviewed drafts of this paper. AR led the data collection and analysis in Madagascar and reviewed drafts of this paper. FS led the data collection and analysis in Ghana and reviewed drafts of this paper. SK led the data collection and analysis in BF and reviewed drafts of this paper. MK led the data collection and analysis in DRC and reviewed drafts of this paper. DP provided input to the framework for analysis of this study, provided technical assistance to the data collection in Ghana and Malawi and reviewed drafts of this paper. All authors read and approved the final manuscript.

## Pre-publication history

The pre-publication history for this paper can be accessed here:

http://www.biomedcentral.com/1471-2458/12/1071/prepub
